# A Common Epitope in p53 and Tau Protein Aggregates

**DOI:** 10.1002/alz.087744

**Published:** 2025-01-03

**Authors:** David Lynch, Nemil Bhatt, Madison Samples, Madeline Demny, Phanourios Tamamis, Rakez Kayed

**Affiliations:** ^1^ University of Texas Medical Branch, Galveston, TX USA; ^2^ Texas A&M University, College Station, TX USA

## Abstract

**Background:**

The oligomers and fibrils of tau are well known as an indicator of Alzheimer’s disease (AD). Recently, other protein aggregates have been shown to be potentially involved in the development of the disease.

One of these proteins is p53, involved in DNA repair. Prior studies in our lab show that p53 will form oligomers and fibrils in AD cases but not healthy controls, and has even been observed as a co‐aggregate with tau protein. Owing to the importance of p53 in mitigating damage from diseased cells, it is possible that loss of function or even misfunction of p53 due to aggregation or mislocalisation could be an early contributing factor to AD, having been shown as a potential early biomarker for AD.

Because of the co‐aggregation of p53 and tau, it is possible that aggregate‐specific antibodies will bind to both p53 and tau. Besides giving mechanistic insights into the proteins’ aggregation, this would also suggest that these antibodies could inhibit aggregation *in vivo*. This study examines this common epitope.

**Method:**

• Using recombinant wild‐type and mutant p53 and tau proteins, the interaction of p53 and tau specific antibodies with each other was determined based around their immunoreactivity, and compared to amyloid‐β.

• Co‐staining of AD mouse model and AD patient samples for both misfolded tau and p53 was used to determine if this conformation cross‐reactivity could also be observed in patient samples.

**Result:**

Despite the lack of sequence homology, an in‐house conformation and aggregation specific p53 antibody was found to be able to react with tau protein, and vice versa. It was also observed that the p53 mutant proteins had different reactions to these aggregate‐specific antibodies. As these antibodies are conformation‐specific, this provides information on the similar conformation both proteins must adopt in order to react with MDM2.

**Conclusion:**

The common epitope recognised by the p53 and tau antibodies suggests that this is a potential target for antibody therapy, as the ability to prevent the formation of the co‐aggregates may permit p53 to continue its role as the ‘guardian of the genome’, a role which, if inhibited, could be a contributing factor to AD.

